# 3D Printing of Alginate-Natural Clay Hydrogel-Based Nanocomposites

**DOI:** 10.3390/gels7040211

**Published:** 2021-11-14

**Authors:** Rebeca Leu Alexa, Raluca Ianchis, Diana Savu, Mihaela Temelie, Bogdan Trica, Andrada Serafim, George Mihail Vlasceanu, Elvira Alexandrescu, Silviu Preda, Horia Iovu

**Affiliations:** 1 Advanced Polymer Materials Group, Department of Bioresources and Polymer Science, Politehnica University of Bucharest, 011061 Bucharest, Romania; rebeca.leu@upb.ro (R.L.A.); andrada.serafim@gmail.com (A.S.); 2 National R-D Institute for Chemistry and Petrochemistry ICECHIM—Bucharest, Splaiul Independentei 202, 060021 Bucharest, Romania; trica.bogdan@gmail.com (B.T.); elviraalexandrescu@yahoo.com (E.A.); 3 Department of Life and Environmental Physics, Horia Hulubei National Institute of Physics and Nuclear Engineering, 077125 Magurele, Romania; savu_diana@yahoo.com (D.S.); mihaela.temelie@nipne.ro (M.T.); 4 Faculty of Medical Engineering, University Politehnica of Bucharest, Gheorghe Polizu 1-7, 011061 Bucharest, Romania; vlasceanu.georgemihail@yahoo.com; 5 Institute of Physical Chemistry “Ilie Murgulescu”, Romanian Academy, Splaiul Independentei 202, 060021 Bucharest, Romania; predas01@yahoo.co.uk; 6 Academy of Romanian Scientists, Splaiul Independentei 54, 050094 Bucharest, Romania

**Keywords:** alginate, hydrogel, natural clay, composites, 3D printing

## Abstract

Biocompatibility, biodegradability, shear tinning behavior, quick gelation and an easy crosslinking process makes alginate one of the most studied polysaccharides in the field of regenerative medicine. The main purpose of this study was to obtain tissue-like materials suitable for use in bone regeneration. In this respect, alginate and several types of clay were investigated as components of 3D-printing, nanocomposite inks. Using the extrusion-based nozzle, the nanocomposites inks were printed to obtain 3D multilayered scaffolds. To observe the behavior induced by each type of clay on alginate-based inks, rheology studies were performed on composite inks. The structure of the nanocomposites samples was examined using Fourier Transform Infrared Spectrometry and X-ray Diffraction (XRD), while the morphology of the 3D-printed scaffolds was evaluated using Electron Microscopy (SEM, TEM) and Micro-Computed Tomography (Micro-CT). The swelling and dissolvability of each composite scaffold in phosfate buffer solution were followed as function of time. Biological studies indicated that the cells grew in the presence of the alginate sample containing unmodified clay, and were able to proliferate and generate calcium deposits in MG-63 cells in the absence of specific signaling molecules. This study provides novel information on potential manufacturing methods for obtaining nanocomposite hydrogels suitable for 3D printing processes, as well as valuable information on the clay type selection for enabling accurate 3D-printed constructs. Moreover, this study constitutes the first comprehensive report related to the screening of several natural clays for the additive manufacturing of 3D constructs designed for bone reconstruction therapy.

## 1. Introduction

Alginate or alginic acid sodium salt is one of the most commonly used anionic polysaccharides for the fabrication of scaffolds, envisioned for tissue engineering. Biocompatibility, bioavailability and the ease of gelation make alginate an ideal candidate for mimicking the morphology of the physiological extracellular matrix [1,2].

In recent years, alginate-based hydrogels were frequently used in the 3D(bio)printing process of specially designed scaffolds [3,4,5,6,7]. Through 3D printing, or the additive manufacturing technique, it is possible to fabricate accurate and reproducible, three-dimensional, alginate-based architectures by facile and material-saving layer-by-layer deposition, otherwise unattainable using conventional manufacturing techniques.

However, designing a suitable alginate-based hydrogel for 3D manufacturing is a real challenge. In order to obtain alginate formulations, the viscosity of alginate hydrogels is the crucial starting point as it is strongly related to the printability and stability of the printed constructs. Sodium alginate solutions are considered non-Newtonian fluids with low viscosities, inadequate for use as a single-component printing ink [8,9,10]. The 3D-printed alginate constructs present a low form fidelity and are unstable in physical conditions. Moreover, increasing the concentration of alginate has no beneficial effect on cell viability and proliferative capacity [2,3,4].

To overcome these problems, different approaches were used, such as the pre-crosslinking of alginate printing formulations, blending alginate with natural or synthetic polymers, compounding alginate inks with different inorganic fillers or the simultaneous use of these methods [11,12,13,14]. These approaches were demonstrated as relatively successful solutions, and improvements in the mechanical performance and biofunctionality of 3D-printed, alginate-based scaffolds were registered.

With respect to the compounding route, nanoclays were widely used for the development of injectable biomaterials, among different inorganic fillers [15,16,17,18,19,20,21]. These hydrophilic, layered silicates have the advantages of high surface area, tailorable surface chemistry and good biocompatibility [22,23,24]. Moreover, their outstanding ability to modulate flow properties, combined with their remarkable capacity to stabilize suspensions and emulsions, are excellent features of interest in the design and development of 3D-printing nanomaterials [19,20,24,25]. The inclusion of nanoclays in the printing inks increases stiffness and scaffold stability, but also has a beneficial influence on the cell adhesion, proliferation and osteogenic differentiation of preosteoblasts [17,20,24,25,26,27]. When necessary, layered silicates can be organomodified with long alkyl chain quaternary ammonium salts to improve their interfacial adhesion with more hydrophobic monomers or polymers [18,22,28,29,30,31,32]. Nanoclays have great potential for the entrapment and controlled delivery of polar/unpolar bioactive agents [22,33,34,35,36].

In recent years, the number of studies referring to the involvement of clays in the 3D-printing process increased. However, as indicated by a recent literature survey which takes into account 3D print and clay/layered silicate/laponite/natural clay/montmorillonite/bentonite, additive manufacturing and clay/layered silicate/laponite/naturalclay/montmorillonite/bentonite as keywords, the number of relevant research studies did not exceed 50 articles. Among these research studies, only seven papers focused on alginate hydrogel-based inks that contained clay; six of them referred to 3D composite constructs envisaged for tissue engineering. Thus, alginate was studied in combination with methylcellulose [17,26], PEGDA/gelatin [37], methylcellulose/polyvinylidene fluoride [38] or hyaluronic acid/polyvinylidene fluoride [39]. Four studies used disc-shaped, synthetic clay-Laponite [17,25,26,39], while two studies used halloysite nanotubes as the inorganic components embedded in the printing formulations [38,39]. Only one study used natural clay, namely, Cloisite 20A, but the 3D-printed constructs were not used for developing scaffolds for tissue engineering but for removing heavy metal ions from wastewaters [40].

In this respect, the present study evaluates the possibility of obtaining natural mineral clay–alginate formulations suitable for use in the extrusion 3D-printing process of composite scaffolds. The mineral clay used in the present study is called montmorillonite, with a general formula [(Si_12_Mg_8_O_30_(OH)_4_(OH_2_)_4_·8H_2_O] which belongs to smectite group [22,37,41]. These types of clays are 2:1 layered silicates, each layer (~1 nm thickness) consisting of one octahedral sheet of alumina sandwiched by two tetrahedral sheets of silicon [18]. As mentioned above, sodium ions from the interlayer space may be exchanged through an ion exchange reaction with cationic surfactants, in order to increase clay compatibility with organic molecules. The resulting amphiphilic clay layers can dissociate in polar or unpolar solvents, forming individual layers which could further generate intercalated and/or exfoliated structures in polymer matrices [18,34,35,36].

Accordingly, the specific structure of the natural organomodified clays used in our study could provide a selective interaction with alginate polymer networks. A systematic comparison between alginate-based inks containing different types of natural clays is absolutely necessary to assess the most suitable natural clay type in relation to printing formulations and the final properties of scaffolds. Therefore, present study assesses the printability, physico-chemical properties and biocompatibility of five alginate-based composite formulations and their 3D-printed constructs and establishes the importance of clay type selection at a fixed concentration.

The present study provides valuable information and results for the applications of natural resources, namely, mineral clay and brown seaweed extract, in the additive manufacturing process of nanocomposite scaffolds intended for personalized regenerative medicine. As far as we know, this study constitutes the first systematic report related to the screening of natural clay in the 3D printing of implants designed for bone reconstruction therapy.

## 2. Results and Discussion

### 2.1. Structure of Nanocomposite Samples—ATR-FTIR, XRD and TEM Analyses

**Figure 1:**
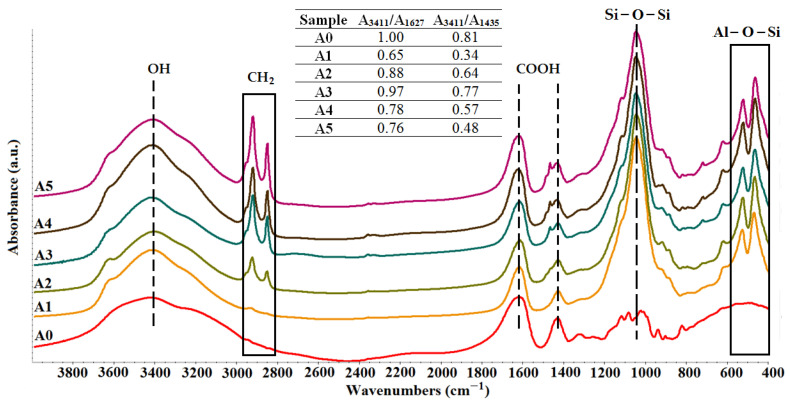
FTIR analysis of commercial alginate (**A0**) and of the synthesized alginate-clay nanomaterials: alginate–Cloisite Na (**A1**), alginate–Cloisite 30B (**A2**), alginate–Cloisite 93A (**A3**), alginate–Cloisite 20A (**A4**), alginate–Cloisite 15A (**A5**).

The FTIR analyses, presented in Figure 1, demonstrated the inclusion of the clay in the alginate matrix by the presence of the particular peaks of clay in the nanocomposite samples. Therefore, the peaks found at 1040–1048 cm^−1^ correspond to the Si-O-Si stretching vibration, while the peaks from 460 cm^−1^ and 520 cm^−1^ come from the Al-O-Si vibration. Peaks around 2856 cm^−1^ were assigned to the asymmetric and symmetric stretching vibration of methylene groups. The methylene groups originated from the organomodifiers agents, namely, hydrocarbon chains of the quaternary ammonium salts [16,31,33]. Another specific peak of modified clays was found at 1450 cm^−1^ and was attributed to the bending vibration of quaternary ammonium located in the interlayer’s clay space [42]. The distinct peaks of organomodifiers were not detected for A1 composite samples, which contained an unmodified clay: Cloisite Na.

Carboxylate and hydroxyl groups from the alginate structure and hydroxyl groups from the clay structure possess a very high interaction probability. However, FTIR spectra revealed that the addition of mineral clays had a very small influence on the peak position of the characteristic absorptions of alginate. Instead, there were changes in the intensities of the alginate-specific peaks. In this regard, the relative intensities of the alginate-clay nanocomposites were calculated as the ratio between the absorption found at 3411 cm^−1^ due to the stretching vibration of the hydroxyl groups and the absorptions from 1627 cm^−1^ and 1435 cm^−1^ which were specific peaks attributed to the asymmetric and symmetric stretching of carboxylate, respectively (the table from Figure 1). The results indicated a decrease in the relative intensity values which points to the hydrogen bonding between the Si-OH/Al-OH groups of the tetrahedral and octahedral sheets of clays and carbonyl groups from alginate. The results were fairly consistent with those obtained by Zhang et al. [41] and Abdollahi et al. [43].

**Figure 2:**
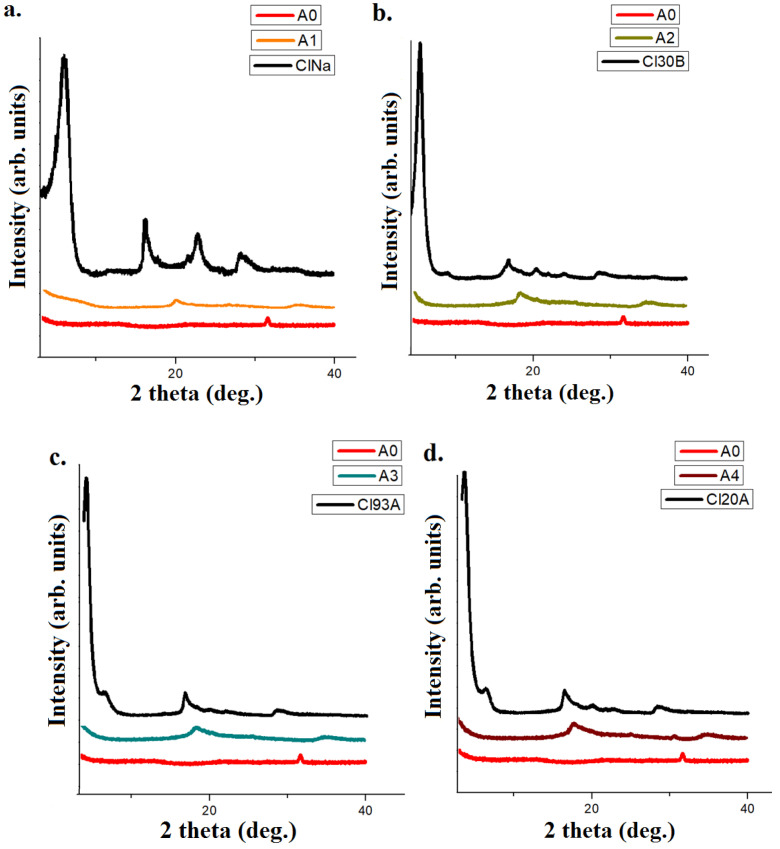

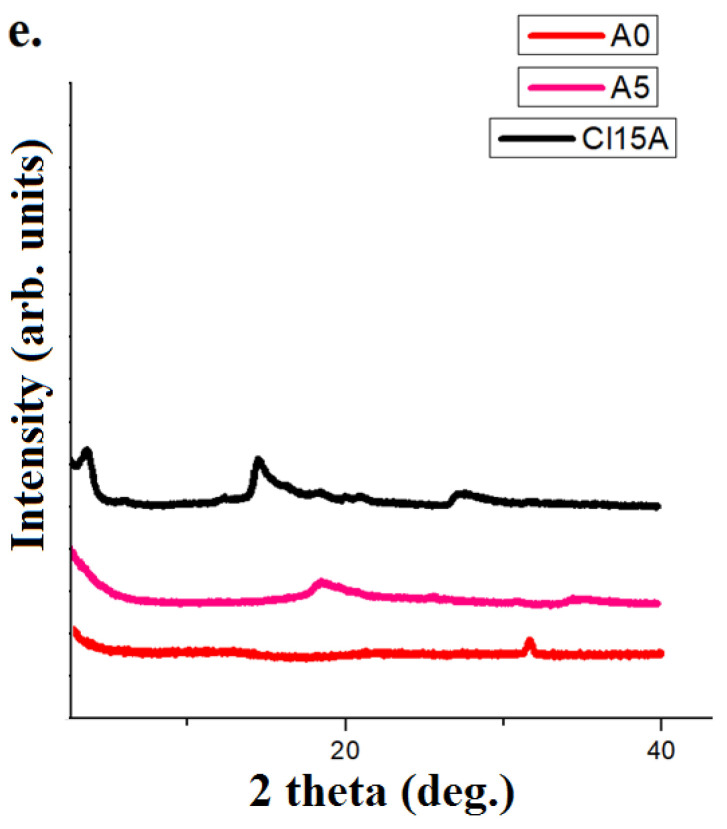
X-ray diffractograms of: (**a**) Cloisite Na, crosslinked alginate–Cloisite Na and alginate matrix; (**b**) Cloisite 30B, crosslinked alginate–Cloisite 30B and alginate matrix; (**c**) Cloisite 93A, crosslinked alginate–Cloisite 93A and alginate matrix, (**d**) Cloisite 20A, crosslinked alginate–Cloisite 20A and alginate matrix and (**e**) Cloisite 15A, crosslinked alginate–Cloisite 15A and alginate matrix.

The X-Ray diffractograms displayed in Figure 2, indicated that the layered silicates were very well dispersed in the amorphous alginate matrix. This fact was indicated by the shifting and decreasing intensity of the peak found at 2 theta = 2–5°, generally attributed to the interbasal distances between the clay layers [22,34,35,44].

**Figure 3:**
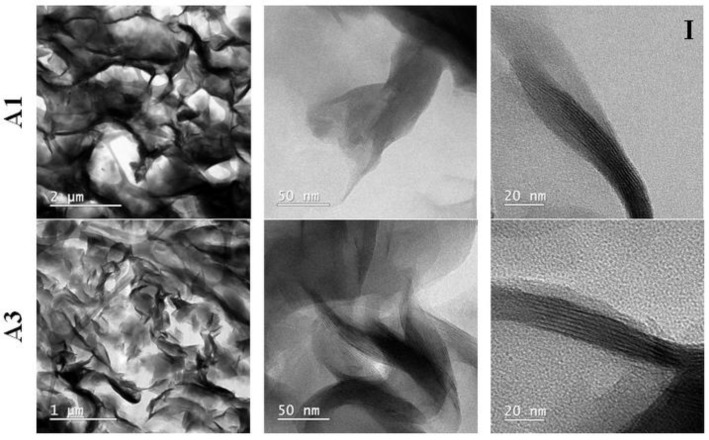

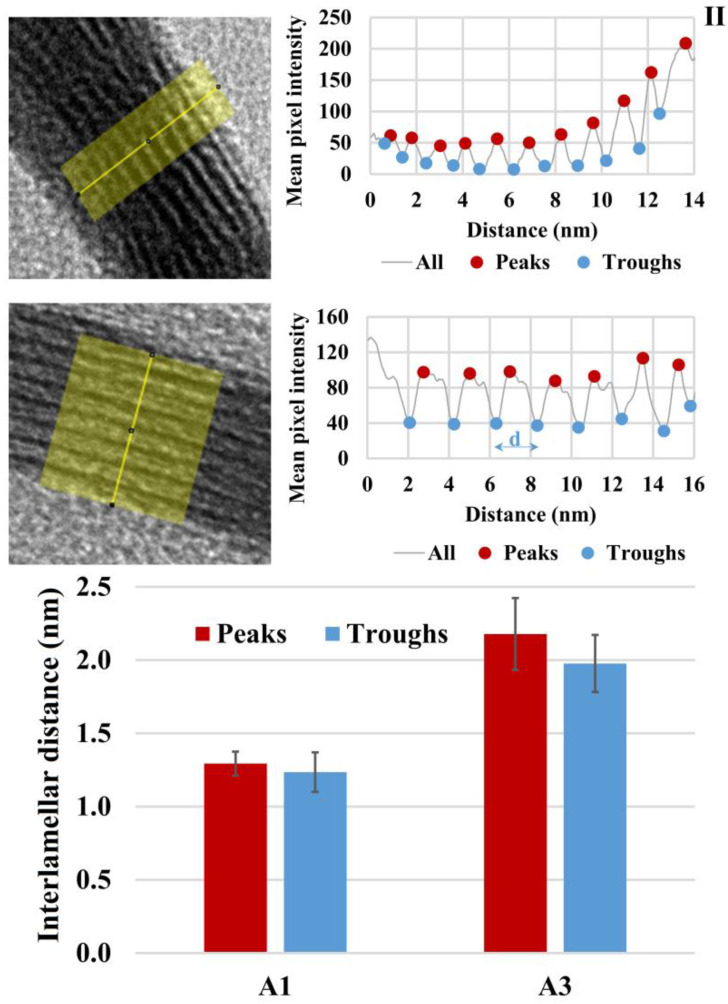
(**I**) TEM images obtained for alginate-Cloisite Na and alginate-Cloisite 93A nanocomposites; (**II**) Analysis of interlamellar distances.

The alginate-based samples compounded with Cloisite Na and Cloisite 93A, were selected for TEM analyses. TEM images depicted a mostly intercalated state of the clay structures, where aggregate comprised of multiple clay layers are dispersed uniformly and randomly into the alginate matrix (Figure 3I).

The TEM images were processed in Fiji Image J and valuable information was extracted (Figure 3II). The clay structures at the nanoscale appear as parallel, alternating black and white lines. A line profile of pixel greyscale values can be plotted perpendicularly to the laminar structures. This allows the distances between the detected peaks and troughs, which correspond to the interlamellar distances observed in the selected samples, to be locally measured. The figure below shows the results obtained for both samples. The mean values are plotted with CI95% bars. There is a clear difference between the distance measured in sample A1 and sample A3 with A3 > A1, pointing to a better compatibility and dispersion of Cloisite 93A clay in the biopolymeric matrix, with respect to the nanocomposites obtained with the Cloisite Na clay type.

### 2.2. Rheological and Mechanical Analysis

**Figure 4:**
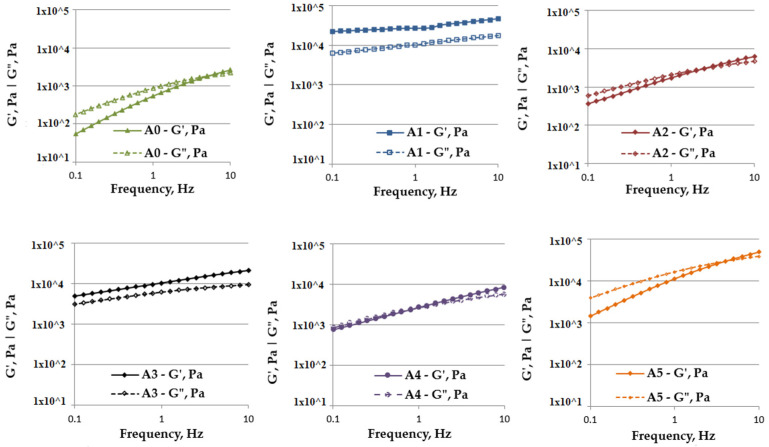
Rheological behavior of alginate-based, 3D-printing inks: **A0**—alginate; **A1**—alginate–Cloisite Na; **A2**—alginate–Cloisite 30B; **A3**—alginate–Cloisite 93A; **A4**—alginate–Cloisite 20A and **A5**—alginate–Cloisite 15A.

The fluid and solid-like behavior of the inks was assessed through oscillatory tests performed at a fixed strain of 1%. While the high frequencies corresponded to the mixing and extruding of the compositions, the low frequencies were attributed to the setting phase. The alginate control sample showed a solid-like behavior only at high frequencies (5–10 Hz), but at lower frequencies a fluid-like behavior is predominant (Figure 4). The investigations revealed that the addition of any clay modified the behavior of the neat alginate matrix. The most obvious influence on the internal structure of the compositions, and therefore on their rheologic behavior, was observed when Cloisite Na and Cloisite 93A were added. In these cases, the solid-like behavior of the compositions was maintained for the whole of the studied frequency range, with a G′ > G″. The addition of clays Cloisite 30B, 20A and 15A caused a slight modification to the rheologic properties of the synthesized inks, lowering the cross-over point to 4 Hz in the case of A5, 2.5 Hz in the case of A2 and 0.8 Hz in the case of A4. Considering that the cross-over point is the inverse of the relaxation time, these modifications indicate that compositions A2, A4 and A5 require longer relaxation times when compared with the A0 control sample, while A1 and A3 maintain their solid-like behavior throughout the whole process.

**Figure 5:**
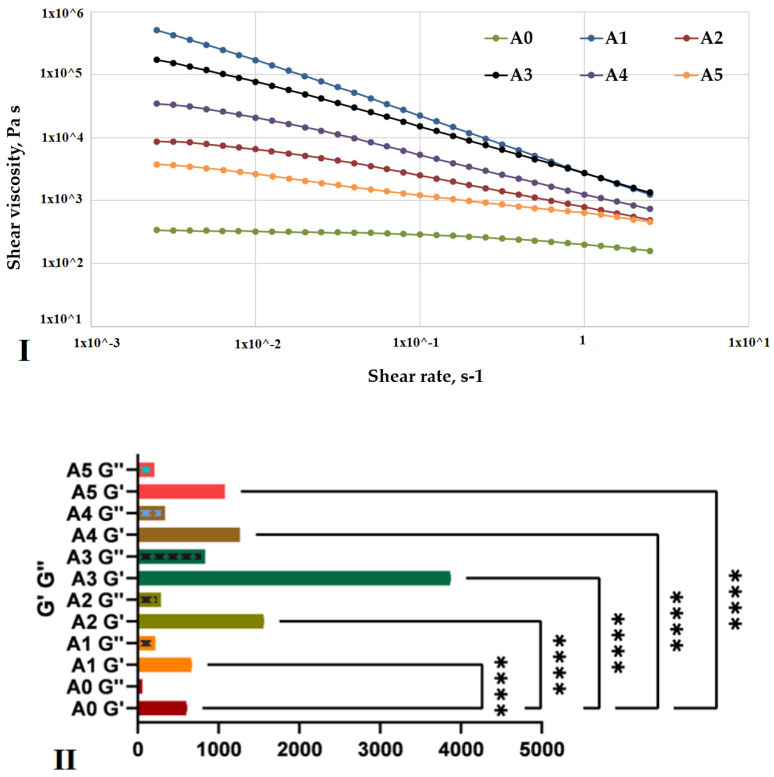
(**I**). Shear viscosity versus shear rate of alginate-based inks at 37 °C; (**II**). Storage and loss moduli determined by nanoindentation on equilibrium, swollen, crosslinked, alginate-based samples. Statistical significance: **** *p* < 0.0001.

The steady shear viscosity measurements showed that the addition of any clay caused significant modifications in the viscosity of the compositions (Figure 5I). While the alginate control sample exhibits a Newtonian flow in the interval 3 × 10^−3^−10^−1^ followed by a slight shear thinning behavior, the addition of clay leads to a predominant non-Newtonian behavior with a pronounced shear thinning effect. The most noteworthy effect on the composition behavior is exhibited by the addition of the clays Closite Na and Cloisite 93A, corresponding to samples A1 and A3, respectively.

Mechanical studies showed that, with the inclusion of the clay in the alginate matrix, the mechanical properties of the nanocomposite samples were improved (Figure 5II). The sample containing Cloisite Na and modified nanoclay presented a higher elastic modulus G′ than samples obtained from pristine alginate. Additionally, all the samples presented a storage modulus G′ higher than loss modulus G″.

All of these data indicate improved mechanical properties; first, it was possible to observe a stronger gel structure after the clay was added to the pristine alginate because the nanoclay layers acted as an elastic solid under stress conditions; secondly, because the samples exhibited an elastic character having G′ > G″, a complete crosslinking process was demonstrated. These results were in good agreement with the data obtained in the literature, which showed that the introduction of clay in polymeric matrices positively influenced the rheological and mechanical behavior of the synthesized nanocomposites.

### 2.3. Swelling and Degradation Studies

**Figure 6:**
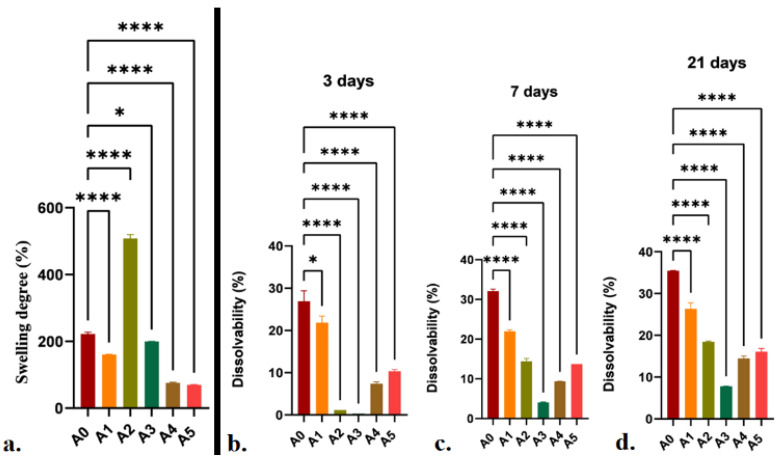
Swelling (**a**) and dissolvability (**b**–**d**) analyses of the alginate-based, 3D-printed scaffolds. Statistical significance: * *p* < 0.05, **** *p* < 0.0001.

When mineral clays were incorporated into the alginate matrix, the hydrophilicity of the alginate changed. When compared to the biopolymeric matrix, all nanocomposite samples displayed varying degrees of hydrophilicity (Figure 6a). In most situations, the degree of swelling diminished when nanoclays were added to the alginate matrix. This phenomenon could be attributed to the following factors: Firstly, the chemical composition of the hydrogel, considering alginate-clay hydrogel bonding and/or electrostatic interactions, occurred. Secondly, the physical barrier effect was induced by the clusters formed by the clay layers, which resulted in limited hydrogel swelling [16,41]. Additionally, the presence of clay organomodifiers, which are quaternary ammonium salts of fatty acids, could lower the sensitivity to water of the nanocomposite scaffolds, thus causing a decrease in swelling [31].

Dissolvability studies indicated that, when clay is added, printed constructs are more stable in physiological, simulated conditions (Figure 6b–d). The capacity of clay layers to generate strong hydrogen bonds with the alginate matrix and clay–alginate electrostatic interactions, reasonably demonstrate the displayed behavior.

### 2.4. Printability of the Materials

Since the major goal of this research study was to develop a nanocomposite printing ink that could be employed in tissue engineering, six inks were evaluated to build biocompatible scaffolds that could allow cells to attach and proliferate. These inks were made from alginate hydrogel, which has biocompatibility and biodegradability qualities. alginate ink, on the other hand, has a low viscosity, which causes the filaments to collapse, and the 3D-printed scaffolds to be unstable. Five types of mineral clays were added to the alginate matrix as possible candidates to increase alginate features such as viscosity, porosity, cell adhesion, and mechanical properties, resulting in new nanocomposite, hydrogel–clay, printable inks. The direct dispensing printhead of the 3D DiscoveryTM bioprinter from RegenHU Ltd., Villaz-St-Pierre, Switzerland, was used to study the printability of the nanocomposite hydrogels. Extrusion technology was used in the direct dispensing printhead. This method allowed for the testing of various materials in a cell-friendly environment because the extrusion process occurred at an ambient temperature. A 3 mL cartridge with metallic and variable needle diameters was used for the extrusion operation (0.25 mm, 0.33 mm, 0.41 mm, 0.65 mm). Parameters such as pressure (150–600 kPa) and printing rates (1.2 mm/s to 11 mm/s) were also investigated in order to find the best fit for each material. Layer-by-layer, BioCAD software was utilized to create 3D structures. BioCAD is a software application that allows you to model items in 2D and then examine them in 3D after the G code is generated.

**Figure 7:**
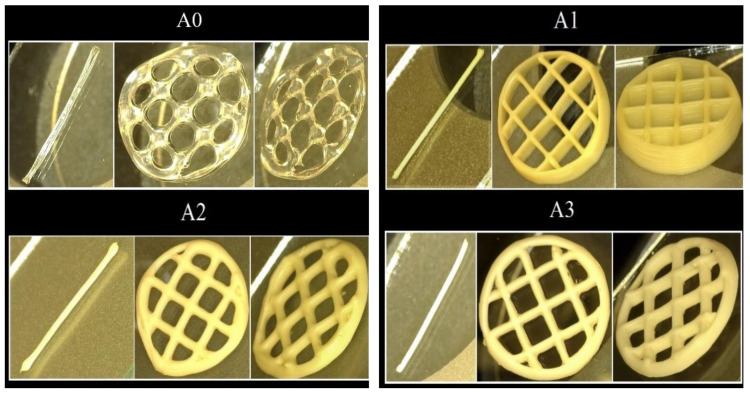

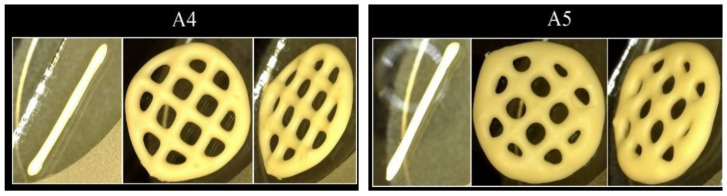
3D-printed scaffolds obtained through the additive manufacturing process of alginate-based inks. Parameters used for each hydrogel based ink: (**A0**): presure 150 kPa, printing rate 11 mm/s, needle diameter 0.25 mm; (**A1**): presure 470 kPa, printing rate 2 mm/s, needle diameter 0.33 mm; (**A2**): presure 250 kPa, printing rate 10 mm/s, needle diameter 0.41 mm; (**A3**): presure 500 kPa, printing rate 4 mm/s, needle diameter 0.41 mm; (**A4**): presure 170 kPa, printing rate 9 mm/s, needle diameter 0.33 mm; (**A5**): presure 190 kPa, printing rate 6 mm/s, needle diameter 0.41 mm.

Because of the low viscosity of alginate and alginate treated with Cloisite 30B, 20A, and 15A, the printing process causes instability, resulting in uneven filaments in the finished scaffolds. Furthermore, the nanocomposites filaments collapsed, and the final scaffolds did not keep their initial dimensions due to the unstable materials (Figure 7). Only five layers could be printed with the mentioned above formulations and when the number of layers increased the printed structure collapsed. Instead, alginate—Cloisite Na and alginate—Cloisite 93A formulations were printed with high accuracy up to 10 layers, presented regular filaments and maintained their initial 3D-printed structure. Moreover, in the case of Cloisite Na up to 20 layers were achieved without collapsing. The 3D-printed scaffolds maintained their initial shape where layers could be very easily distinguished (Figure 7A1).

In summary, the recently created nanocomposites inks, based on alginate and Cloisite Na or Cloisite 93A, were the best formulations for allowing the printing of stable filaments and 3D constructs with remarkable shape fidelity and well-ordered surfaces compared to pure alginate and other composite samples.

### 2.5. Scanning Electron Microscopy (SEM) and Micro-CT Analyses

**Figure 8:**
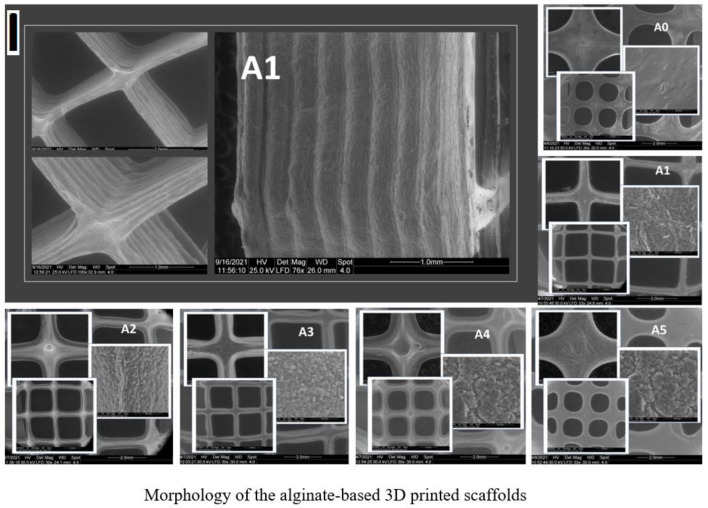

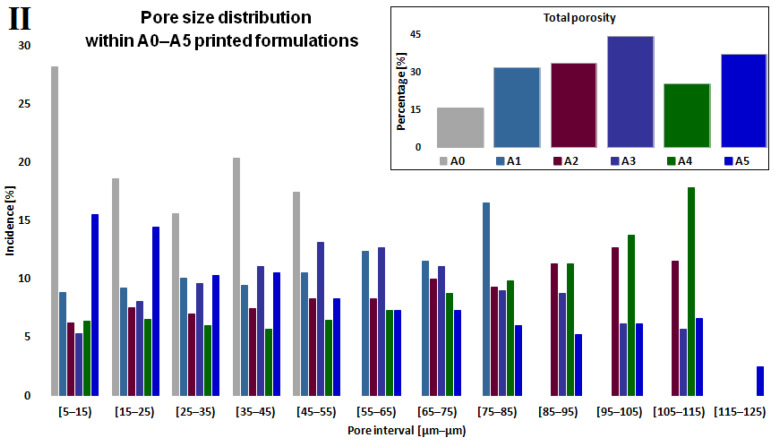
(**I**) Morphology of the alginate-based, 3D-printed scaffolds; (**II**) Quantitative results obtained in CTAn (Bruker) with respect to pore size distribution of the 3D-printed scaffolds.

All composite samples presented a roughness when compared with pristine alginate samples, as indicated by SEM images (Figure 8I). Analyzing the roundness of open pores could provide a way to quantify the printed shapes. The average roundness of the open pores of the 3D-printed scaffolds was calculated using SEM images and the Wadell equation [45]. R = 1 is attained for a completely round object; however, irregular shapes tend to have values smaller than 1. The pristine alginate sample had the greatest calculated value of 0.76, whereas alginate Cloisite Na and alginate Cloisite 93A had the lowest R values—0.23 and 0.2, respectively. These significant differences suggest that, in the presence of Cloisite Na and Cloisite 93A, 3D-printed constructions maintained their shape better. This fact supports the reality observed in the 3D-printing process, as well as in the evaluation of the stability of the printed forms generated when Cloisite Na and 93A were used in composite formulations.

Moreover, the compositions with clays generated different outcomes with respect to freeze-drying porogenesis, as plotted in the charts from Figure 8II. The porosity ratio in the materials was generally lower than expected when using the fabrication method. In the inset from Figure 8II, the total porosity of each object is depicted, with respect to the total volume of the print, minus the square-shaped inlays of the CAD model. A0 features a total porosity of only 15%, which might be attributed to the high solid content of the alginate precursor material (10%wt.). This ratio and the strong affinity between the polymer chains concur for this low level of porosity, which might be a disadvantage of optimal cell infiltration and proliferation, as the available surface for adhesion and interconnected channels are poor (Figure 8I).

Nonetheless, upon clay addition, the composite network is more susceptible to allowing the generation of more porous architectures. The biphasic nature of the A1–A5 materials is an advantage in the first steps of freeze-drying fabrication, supporting the solid matter separation from the solvent upon freezing. The additional interaction clays forge with the macromolecular network enables and enhances the nucleation of the frozen solvent centers and the propagation in volume. This behavior is mirrored in the total porosity calculated in CTAn (Figure 8I) which is superior for all composite series compared to the A0 control.

Additionally, the pore size distribution is drastically influenced by clay addition. The control has the narrowest pore distribution, with most of the pores prevailing in the 5–15 µm domains. The clay addition determines the broadening of the pore size. A5 pores also occur preferentially in the smaller domains, as the incidence of larger concavities decreases linearly; however, the empty space channels also cover large values. The pore distribution in the A3 formulation best emulates the Gaussian distribution while, in the case of A4, the incidence of the larger pores is superior. Such architectures might be the best choices when dealing with cell cultures, since they must have adequate interconnected networks of sufficient magnitude in order to successfully penetrate through the scaffold for optimal population.

This variability of internal pores is reflected by the iv. subdivisions of Figure 8I, A0–A6; the A0-printed structures seem virtually pore-less and fully compact due to the fact that, overall, the porosity is low, and the pores are very small, mostly in the vicinity of the metric equivalent of scanning resolution. Nonetheless, the clay reinforcement impacts this morphological characteristic and larger pores emerge, as seen in the marked boxes in Figure 8I.

**Figure 9:**
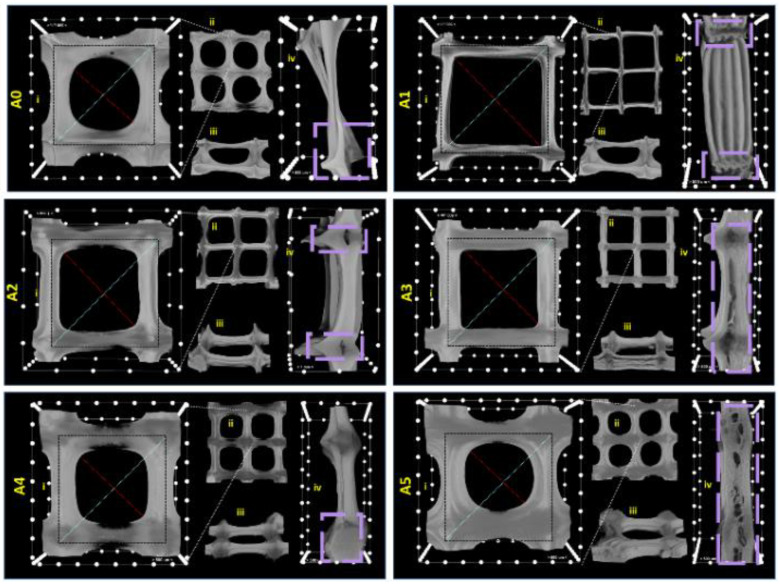
Multi-angle depiction of 3D-printed (**A0**–**A5**) inks by means of micro-CT analysis: (**i**) close-up on basic square lattice of the 3D model; (**ii**) Frontal view of four-square units of the print; (**iii**) Side view of the object surfaces; (**iv**) Lateral cross-section illustrating inner architecture of the freeze-dried material. In the i. and iv. images, the scale unit is 500 µm. The blue and red lines mark the inner and outer extremities of the printing unit while the violet-dotted perimeters highlight the porosity of the printed objects, from the cross-sectional view.

The xerogels obtained after freeze-drying the 3D-printed objects exhibit a distinct morpho-metrical behavior post-deposition from the extrusion needle, as illustrated in Figure 9. The fidelity of the resulting printed constructs compared to the square lattice CAD model varies the function of the inorganic filler type, which reinforces the polymeric matrix. Generally, the addition of clays seems to improve the shape stability of the scaffolds, as indicated from the preservation of the 90° angles of the printing unit’s design (Figure 9i,ii) and the distinction between the adjacent layers in the cross-sectional view (Figure 9iv).

Empirically, the A1, A2, and A3 compositions seem to provide the best rheological support for preserving the shape of the desired print, which could be attributed to the favorable OH bonding interactions between the major phases with the specific clays that tune the viscosity of the inks [41,46]. Moreover, the object fabricated from the A1 composition provides the best stability, as per the clear delineation of each deposited layer (Figure 9A1(iv)) and the smoother surface (Figure 9A1(iii)) compared to the rest for the batch. Additionally, in the case of A4 and A5, the square pattern is also sustained, despite the fact that the first deposited layers manifested a tendency to collapse and spread post-deposition. These variations can be observed from the difference between the inner and outer extremities (diagonal axis) length. Therefore, by comparison to the control (Figure 9A0), clay addition endorsed the preservation of the desired pattern.

### 2.6. Biological Analyses

To evaluate the in vitro cytotoxicity of the investigated alginate (A) and alginate combined with different types of clay (A1, A2, A3, A4, A5) we assessed the MTT tetrazolium salt viability and then used supernatants of normal L929 fibroblasts cells to measure LDH release from the damaged cells exposed at the undiluted and diluted extracts of the composites for 24 h. All the extracts were clear and had a pH of 7–8.

**Figure 10:**
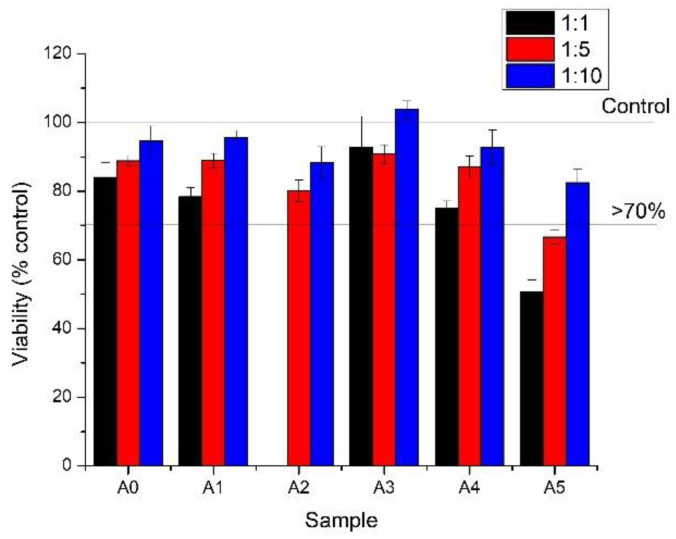
Biocompatibility evaluation of alginate samples on L929 cells by MTT viability assay at 24 h of incubation with samples extracted in culture media (1:1) or dilutions of 1:5 and 1:10 of the extracts.

**Figure 11:**
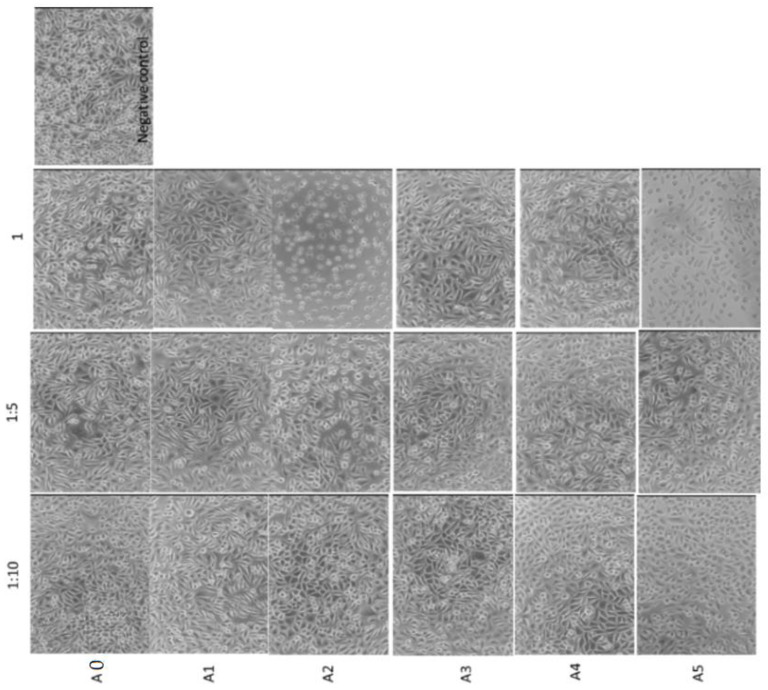
L929 cell culture optical microscope images following 24 h of incubation with alginate extracts.

According to MTT test, the most undiluted or diluted extracts induced a higher viability than 70%, which means that the samples were biocompatible (Figure 10). The only exceptions were represented by undiluted A2 and A5 extracts which exhibited high cytotoxicities. These results are strenghtened by the morphologies and densities of the cells observed under the light microscope (Figure 11). The cells appeared to be modified (round and detached) only in the case of treatment with undiluted A2 and A5 extracts.

**Figure 12:**
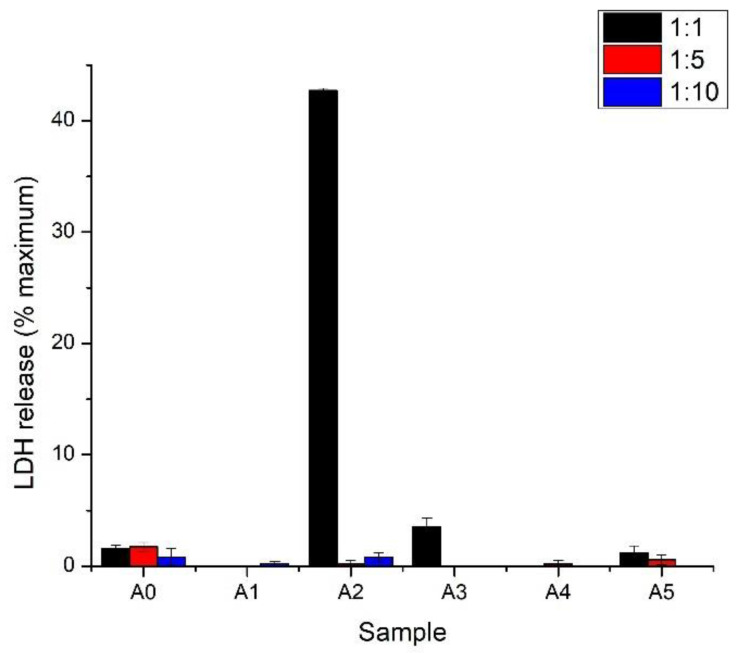
Biocompatibility evaluation of alginate samples on L929 cells by LDH release at 24 h of incubation with samples extracted in culture media (1:1 or 1:5 and 1:10 dilutions of the extracts).

As indicated by the LDH release assay, all the samples’ extracts seemed to be noncytotoxic with the exception of undiluted A2 extracts, which induced a release of 40% of LDH in the L929 culture media (Figure 12). This outcome implied that the lysis of cells was produced by affecting the plasma membrane integrity.

Most of the samples presented a good biocompatibility in L929 cultures, even when using undiluted extracts (A0, A1, A3, A4) with >70% viability. Among the extracts mentioned, we found no significant changes. However, A2 and A5 presented a high cytotoxic effect on fibroblasts culture when used at 100% concentration. Diluted extracts lead to a lower effect on cellular viability of the cells for all extracts.

At undiluted extracts (1), some of the samples (A2 and A5) had a cytotoxic effect, characterized by the rounding and detachment of the cells. The other extracts (A, A1, A3 and A4) had no notable effect on L929 cultures. At dilutions of 1:5 and 1:10, no cytotoxic effect was observed at the level of the morphology and density of cell cultures.

Most of the extracts (A0, A1, A3, A4, A5) did not induce cellular lysis and the subsequent release of LDH in the culture media of L929 cells. Only A2 sample extracts presented a high cytotoxic effect on fibroblast cultures when used at 100% concentration, leading to an approximate LDH release of 40%. However, the diluted extract did not induce LDH release.

Therefore, all alginate-clay composite hydrogels showed good cell viability, with hydrogels with Cloisite Na and Cloisite 93A presenting the best biological results. The obtained results are in full agreement with previous studies that showed a good biocompatibility of the obtained composites by the inclusion of clay in polymeric matrices [38,47]. The differences between clay types could be related to specific clay concentrations but also with nature of the organomodifier in the clay, which could regulate the toxicity of the functionalized clay [48].

Considering the morpho-structural observations of alginate-based formulations and their associated 3D-printed constructs, as well as the outcomes of the 3D-printing process cumulated with biology results, the alginate sample containing Cloisite Na was selected as the most relevant for in depth-biological analyses.

### 2.7. Cells Differentiation and Mineralization

**Figure 13:**
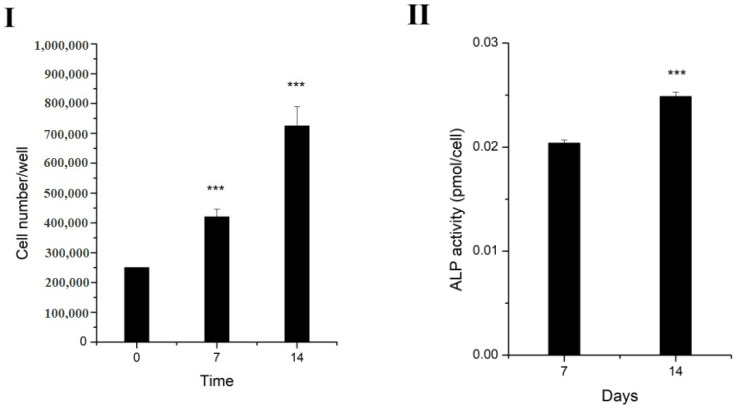
(**I**) Cellular proliferation of MG-63 cells. Cell number increased significantly over time. Values represent mean of at least 4 samples ± SEM. (**II**) Alkaline phosphatase released in the culture media by MG-63 cells grown in normal culture media in the presence/absence of the alginate sample. Bars represent mean of at least 4 samples ± SEM. *** *p* < 0.001.

The evaluation of the proliferation of MG-63 cells (Figure 13I) showed that the cells grown in the presence of the alginate sample containing Cloisite Na were able to proliferate at 7 and 14 days (*p* = 0.007 and *p* = 0.005, respectively, compared to the control; *p* = 0.01 at 14 days compared to the results at 7 days). Trypan Blue staining of the cells indicated no cellular death.

Alkaline phosphatase is an enzyme with several functions throughout the body, including bone formation, where ALP is synthetized by osteoblasts and is involved in the calcification of the bone matrix [49]. The increased production of ALP was found in the media collected from the cells incubated with alginate samples for 14 days, compared to the samples incubated for 7 days (*p* = 1.31737 × 10^−^^7^), suggesting the osteogenic differentiation of the cells without the requirement of chemical inductors (Figure 13II).

**Figure 14:**
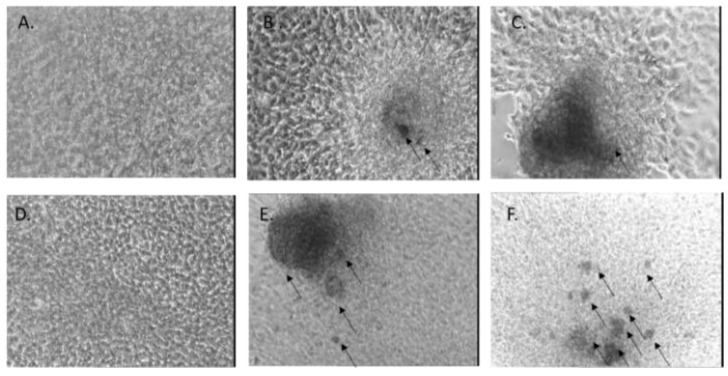
Alizarin Red staining of MG-63 cells grown in normal culture conditions (control) for 7 days (**A**) or 14 days (**D**), or in the presence of alginate samples for 7 days (**B**,**C**) or 14 days (**E**,**F**). Images were obtained using an inverted phase-contrast microscope with a black-and-white camera attached. Dark areas correspond to Alizarin Red staining.

Alizarin Red staining indicated the mineralization process in tissue or cell cultures (represented by calcium deposits). In Figure 14, we can observe that the control cells did not present any staining, indicating that they did not form calcium deposits at 7 or 14 days of culture. alginate-Cloisite Na samples induced the formation of calcium deposits starting from 7 days of culture, when some staining was observed mainly in areas of the surface containing a very dense population of cells. At 14 days of culture, more calcium deposits were observed as spreading to the less-populated area of the slides.

Therefore, the composite sample of the alginate—Cloisite Na, was able to induce the production of calcium deposits in MG-63 cells in the absence of specific signaling molecules. The obtained data were in good agreement with other studies, which proved that polymer–clay composites were suitable materials for cellular proliferation and osteogenic differentiation [50,51].

## 3. Conclusions

Printable nanocomposite inks and further 3D-printed scaffolds, based on alginate and several types of commercial clays, were successfully obtained. Morphological analyses confirmed the increase in porosity with the introduction of several types of clay in the alginate matrix, especially with Closite Na and a selective dispersion of nanoclays within the biopolymeric matrix. Rheological and mechanical analysis demonstrated improved properties with the introduction of clay, particularly compositions A1 (alginate—Cloisite Na) and A3 (alginate—Cloisite 93A). The use of a particular type of clay at a precise concentration significantly influenced the features of the composite inks but also of the printed structures. The 3D-printed nanocomposite scaffolds preserved their shape and presented modified properties as functions of the clay type used in the synthesis process; thus, the samples obtained with the compositions A1 and A3 conferred the best-preserved 3D architecture after printing.

A biological analysis indicated that most of the samples presented a good biocompatibility in L929 cultures while the alginate—Cloisite Na sample was able to induce the production of calcium deposits in MG-63 cells in the absence of specific signaling molecules.

All of these data confirm that the selected alginate—Cloisite Na formulation is a suitable composite ink to produce stable and accurate 3D-printed scaffolds for osteogenic differentiation, and thus bone tissue regeneration.

Future perspectives could include the variation of the concentration of clay, especially Cloisite 93A, for the obtaining of precise biocompatible 3D architectures. Moreover, our future approaches will pursue the inclusion of a bioactive agent directly into the printing formulation in order to modulate its delivery through the addition of clay of different concentrations.

## 4. Materials and Methods

### 4.1. Materials

Alginic acid sodium salt and calcium chloride were purchased from Sigma-Aldrich, Norway. Natural montmorillonite (MMT) and organomodified montmorillonite (OMMT) were offered by Southern Clay Products Inc. (Gonzales, TX, USA) and were used as received. The different types of commercial clays listed are in the order of increasing hydrophobicity, as follows: Cloisite^®^Na—sodium form of montmorillonite (92 meg/100 g); Cloisite^®^ 30B—organomodified with methyl, tallow, bis-2-hidroxyethyl (90 meg/100 g); Cloisite^®^ 93A—organomodified with methyl, dihydrogenated tallow (90 meg/100 g); Cloisite^®^ 20A—organomodified with dimethyl, dihydrogenated tallow (95 meg/100 g); Cloisite^®^ 15A—organomodified with dimethyl, dihydrogenated tallow (125 meg/100 g). Ultrapure water and phosphate-buffered saline (PBS) solution (pH = 7.4) were prepared in our laboratory.

### 4.2. Preparation of the Hydrogel

Nanocomposites-based inks were obtained as follows: 2.4 g clay powder (Closite Na, Closite 30B, Cloisite 93A, Closite 20A and Cloisite 15A) was dispersed under vigorously magnetically stirring (500 rpm) in 9 mL ultrapure water for 24 h at room temperature. Then, the aqueous clay dispersion was ultrasonicated for 2 min with ultrasonic probe. Afterwards, 1 g alginate was added in the clay dispersion, under gentle magnetically stirring. In order to allow the polysaccharide be completely hydrated and to obtain a completely homogenously mixture, the sample was placed in an orbital shaker for 24 h, at 800 rpm at 40 °C. The samples were named as follows: A0—blank alginate, A1—alginate–Cloisite Na, A2—alginate–Cloisite 30B, A3—alginate–Cloisite 93A, A4—alginate–Cloisite 20A, A5—alginate–Cloisite 15A.

### 4.3. 3D Printing of Alginate Nanocomposites-Based Inks

The nanocomposites ink formulations were printed using the 3D bioprinter 3D Discovery™ from RegenHU Ltd., Switzerland, Villaz-St-Pierre. Direct dispensing print-head was used to perform the tests, with a syringe of 5 mL with an attached cylindrical nozzle of 22G (ø 0.41 mm) and different pressures in the range of 70–350 kPa. The printing process was performed at room temperature (25–30 °C). In order to preserve the printed shape, the samples were crosslinked immediately after the 3D-printing process by immersion of the constructs in a 2%wt. CaCl_2_ solution for one hour.

### 4.4. Fourier Transform Infrared Spectrometry with Attenuated Total Reflectance Accessory (ATR-FTIR)

Alginate, natural montmorillonite, organomodified montmorillonite and nanocomposites ink formulations were structurally characterized using Fourier Transform Infrared Spectrometry. The analyses were performed on FTIR spectrometer Vertex 70 Bruker (Bruker, Billerica, MA, USA). FTIR spectra were recorded in the 4000–400 cm^−1^ wave number range. FTIR analyzes were performed quantitatively, using 200 mg KBr and 1 mg sample.

### 4.5. Scanning Electron Microscopy (SEM) and Micro-Computed Tomography (Micro-CT)

To determine the internal structure and morphology of the printed lyophilized scaffolds, an environmental scanning electron microscopy (ESEM-FEI Quanta 200, Eindhoven, The Netherlands), was used. The samples were analyzed as such without being sputter-coated.

Qualitative and quantitative micro-computer tomography investigations were performed with Bruker µCT 1272 high-resolution equipment. Tomograms were reconstructed from the raw data in Bruker NRecon software. Reconstructed tomograms were rendered in CTVox 3.3.0 (Bruker). The visualization software depicts the 3D reconstruction in 256 grey tones, as shown in Figure 9. Figure 9 illustrates the CTVox six 3D dataset volumes in the scaled cutting box, where the distance between two marks is equal to 0.5 mm. Figure 8II depicts the morphological analysis used to quantify the pore size distribution and porosity found in the freeze-dried printed objects.

### 4.6. X-ray Diffraction (XRD)

X-ray Diffractometer (Rigaku Ultima IV, Tokyo, Japan) was used to determine the structure of the nanocomposite samples. ACuKα radiation (λ = 1.5406 Å), operated at 40 kV and 30 mA was used. All the analyses were performed on powder-form samples at room temperature and atmospheric pressure. The scanning speed used was 1°/min and the interval in which the data were collected was 2θ range 1–50°.

### 4.7. Rheological and Mechanical Analyses

The rheological behavior of the compositions was investigated using a Kinexus Plus Rheometer equipped with a Peltier element for precise temperature control and a water lock to prevent solvent evaporation. Plate–plate geometry was used and a fix gap of 0.5 mm was set for all measurements. First, the linear viscous region (LVR) was established and the frequency sweep measurements were performed at a strain of 1% for all samples within the frequency range 0.1–10 Hz. Steady shear viscosity tests were also performed in the shear rate interval 3 × 10^−3^−3 × 10° s^−1^.

Mechanical analysis was performed on the crosslinked samples, using the Nano Indenter^®^ G200 (Santa Clara, CA, USA). Using a G-Series DCM CSM Flat Punch Complex Gel Modulus, the storage modulus G′, loss modulus G″ and complex shear modulus G* were determined.

### 4.8. Swelling Degree, Degradability of the 3D-Printed, Hydrogel-Based on Alginate Nanocomposites

The swelling degrees of 3D-printed scaffolds were measured at certain times, until the samples reached the maximum saturation level.

Degree of swelling was calculated using the following equation:

(1) $$DS\left(\%\right)=\frac{Ww-Wd}{Wd}\times 100,$$

where *Ww* and *Wd* represent the weight of the wet sample and dry sample, respectively.

The dissolvability of the 3D-printed scaffolds was studied at 3 days, 7 days, and 21 days. In order to study the dissolvability, scaffolds were immersed in PBS, and at predefined times the samples were removed from PBS, lyophilized and weighed.

Dissolvability of the samples was calculated using the equation below:

$$D\left(\%\right)=\frac{W0-Wd}{W0}\times 100$$

where *W*0 represents the initial weight and *Wd* represents dry weight.

### 4.9. Biocompatibility Evaluation of Alginate Samples

Biocompatibility of alginate samples was evaluated by using two in vitro cytotoxicity tests: MTT ((3-(4,5-dimethylthiazol-2-yl)-2,5-diphenyltetrazolium bromide) assay (for cell viability) and LDH (Lactate Dehydrogenase) release measurement (for plasma membrane integrity).

#### 4.9.1. Preparation of Alginate Sample Extracts

The cytotoxicity of the alginate compounds was determined by using the extract dilution method. First, the samples were weighted and sterilized by gamma irradiation at a dose recommended for medical materials (25 kGy). Then, each sample was placed in a glass bottle in complete culture media using an extraction ratio of 0.1 g to 1 mL of media. The vessels were placed in a water bath at 37 °C, with agitation at 100 rpm for 24 h in order to obtain an extract of the samples, according to ISO-10933-12 standard describing sample preparation for biological evaluation of medical devices (ISO-10993 12:2012). The media were removed from the samples and centrifuged for 10 min at 5000× *g*. Clear supernatant was transferred to a new tube. Dilutions of 1:5 (20%) and 1:10 (10%) were prepared in complete culture media.

#### 4.9.2. Cell Culture

L929 (400260, CLS, Eppelheim, Germania) fibroblast cells were used for biocompatibility evaluation of alginate samples. Cells were routinely grown in MEM media (Lonza, Basel, Swissland) supplemented with 10% FBS, and maintained in a humidified incubator, at 37 °C, 5% CO_2_. Cells were plated in a 96-well plate at a density of 3000 cells/well 2 days before addition of extracts to allow cells to adhere to the vessels and start growing so that at the time of the experiments they were at ~70–80% confluence, in an exponential growth phase.

#### 4.9.3. MTT Assay

Media were removed from the 96-well plated with L929 cells and 100 µL of alginate sample extracts, or dilutions of the extract were added to each well in triplicate. Cells were incubated for 24 h in the incubator. At the time of analysis, media were removed, and 100 μL of MTT (Promega, Madison, WI, USA) reagent (0.5 mg/mL in complete culture media) was added to each well. Cultures were incubated for 3 h at 37 °C. Media were removed and 100 μL of DMSO were added to each well. Plates were measured by a Mithras Spectrophotometer at 570 nm. Blank (no cells wells) absorbance was subtracted from each sample. Cellular viability was calculated by the formula: (Abs sample/Abs control) ×100.

#### 4.9.4. LDH Assay

LDH release was measured by CyQUANT™ LDH Cytotoxicity Assay (Invitrogen, Waltham, MA, USA)) following manufacturer’s instructions. Media were removed from the 96-well plate with L929 cells and 100 µL of alginate sample extracts, or dilutions of the extract were added to each well in triplicate. Cells were incubated for 24 h in an incubator. A maximum LDH release sample was prepared by the addition of 10 µL lysis solution to 3 wells at 30 min before analysis. An amount of 50 μL of media were transferred to a new plate and 50 μL of LDH reagent were added to each well. Plates were incubated at room temperature for 30 min and 50 μL of stop solution was added to each well. Plates were measured by a Mithras spectrophotometer at 585 nm with a reference of 690 nm. LDH release was calculated by the following formula: [(abs sample−abs control)/(abs maximum LDH release-abs control)] × 100.

### 4.10. Cells Differentiation and Mineralization

#### 4.10.1. Cell Culture

Osteoblast-like cells (MG-63 osteosarcoma cells—ECACC, Salisbury, UK) were used to evaluate the ability of the alginate samples to improve bone regeneration. Cells were routinely grown in MEM media supplemented with 10% FBS Superior, and 1% Non-Essential Amino-Acids (NEAA) (Sigma-Aldrich, Saint Louis, MO, USA), and maintained in a humidified incubator, at 37 °C, 5% CO_2_.

#### 4.10.2. Cellular Proliferation

MG-63 cells were plated on 12-well plates at a density of 250,000 cells/slide. At the same time, alginate samples were placed in culture media in separate wells. The next day, the samples were washed once with culture media and placed on top of the cell layer. Control samples were represented by MG-63 cells grown similarly but without alginate samples. The cells were grown in normal culture media that was changed every 2–3 days. At 7 and 14 days of culture one set of cells were detached and counted with a haemocytometer using Trypan Blue (Sigma-Aldrich, Saint Louis, MO, USA), to exclude dead cells.

#### 4.10.3. Alkaline Phosphatase

Alkaline phosphatase (ALP) production was evaluated using the abcamab83369 Alkaline Phosphatase Assay Kit, following manufacturer’s instructions, applied on culture media collected from cells grown, as described earlier, at 14 days of culture. Measurements were normalized to the cell numbers.

#### 4.10.4. Alizarin Red

Cells were grown similarly on 12 mm glass coverslips. At 7 and 14 days of culture one set of slides were fixed. The samples were washed with PBS, fixated in 10% paraformaldehyde, washed with distilled water, and stained with a 4 mM solution of Alizarin Red (Sigma-Aldrich) Saint Louis, MO, USA) in water, for 45 min, at room temperature, in the dark. Then, the samples were washed 4 times with distilled water, and kept in PBS for visualization. Images were taken using an inverted phase-contrast microscope.

### 4.11. Statistical Analyses

The data are expressed as mean SD. The significance of differences was evaluated by one-way ANOVA. Significance was considered if *p*-value was < 0.05.

## Data Availability

Not applicable.
